# Brisbane Burn Scar Impact Profile for children aged less than 8 years cross-cultural adaptation

**DOI:** 10.1590/0034-7167-2024-0153

**Published:** 2025-08-08

**Authors:** Francieli Ferreira de Andrade Batista, Rosângela Aparecida Pimenta, Elisângela Flauzino Zampar, Renne Rodrigues, Elza Hiromi Tokushima Anami, Maria Elena Echevarría-Guanilo

**Affiliations:** I Universidade Estadual de Londrina. Londrina, Paraná, Brazil; II Universidade Federal da Fronteira Sul. Chapecó, Santa Catarina, Brazil; III Universidade Federal de Santa Catarina. Florianóplolis, Santa Catarina, Brazil

**Keywords:** Translating, Validation Study, Child, Burns, Quality of Life, Traducción, Estudio de Validación, Niño, Quemaduras, Calidad de Vida

## Abstract

**Objectives:**

to cross-culturally adapt the Brisbane Burn Scar Impact Profile for Caregivers of Children aged less than 8 years for use in Brazil.

**Methods:**

a methodological study, following the stages of translation, synthesis of translations, back-translation, judge committee, pilot test, pre-test and sending of the adapted version for approval by the instrument authors. The Content Validity Index (CVI) was used to assess agreement among judges and to assess the understanding of the instrument among caregivers. For the overall score and dimensions of the instrument, Cronbach’s alpha was used.

**Results:**

an CVI ≥0.87 was obtained for each item in the analysis carried out by the expert committee, and in the pilot test, CVI ≥0.80. The overall Cronbach’s alpha was 0.94 for all dimensions, except for friendships and social interaction (alpha ≥0.70).

**Conclusions:**

the cross-cultural adaptation presented adequate results, supporting the validity of the translated version.

## RESUMEN

**Objetivos:**

adaptar transculturalmente el Brisbane Burn Scar Impact Profile for Caregivers of Children aged less than 8 years para su uso en Brasil.

**Métodos:**

estudio metodológico, siguiendo las etapas de traducción, traducción síntesis, retrotraducción, comité de jueces, prueba piloto, preprueba y envío de la versión adaptada para aprobación de los autores del instrumento. Se utilizó el Índice de Validez de Contenido (CVI) para evaluar el acuerdo entre jueces y evaluar la comprensión del instrumento entre los cuidadores. Para la puntuación global y dimensiones del instrumento se utilizó el alfa de Cronbach.

**Resultados:**

se obtuvo un CVI ≥0,87 para cada ítem en el análisis realizado por el comité de expertos, y en la prueba piloto, CVI ≥0,80. El alfa de Cronbach global fue de 0,94 para todas las dimensiones, excepto para amistades e interacción social (alfa ≥0,70).

**Conclusiones:**

la adaptación transcultural presentó resultados adecuados, apoyando la validez de la versión traducida.

## INTRODUCTION

The number of burn cases has been decreasing in recent years worldwide, with a more significant reduction in Brazil, while in other countries, such as India, an increase in this prevalence has been identified^(1)^. Even so, childhood burns continue to be a major challenge for healthcare services and economic policies, as more than half of the years of life are lost due to disabilities resulting from burn accidents^(2,3)^. In general, injuries are highly painful and traumatic, which can produce a significant negative impact on both physical and psychosocial functioning, leading to increased distress, anxiety and trauma, compromising the health-related quality of life (HRQoL) of both patients and their caregivers^(4-6)^.

HRQoL is a concept widely used in public health, and its measurement may be inaccurate in the pediatric population, given the difficulty in understanding and responding to the assessment items. Therefore, parents/caregivers’ perception is essential to assess children’s quality of life^(7)^. Measurement is essential to qualify the burden caused by burn scars in survivors, and healthcare professionals should consider victims’ perspectives on their scars as an important indicator of quality of life^(8,9)^.

Due to the complexity of the care provided to children who are victims of burns, the diversity of cases and the subjectivity of this impact on HRQoL, instruments for assessing this construct are essential for this assessment. However, in Brazil, there are still no specific validated instruments for this purpose, which highlights a gap in knowledge and makes it difficult to measure the impact of burns on HRQoL in children under 8 years of age. A systematic review on the measurement properties of instruments that assess HRQoL in children indicates the Brisbane Burn Scar Impact Profile (BBSIP) as an appropriate tool for this population, especially when compared to the other two instruments available in the literature for this age group^(10)^.

In this way, the BBSIP allows assessing HRQoL in children under 8 years of age, helping to determine the healing burden, functionality, behavioral attitudes and social coexistence after suffering this condition, as well as allowing the effectiveness of interventions throughout the care process to be safely measured^(11)^.

Considering the importance of using patient-reported outcome measures for clinical decision-making, promoting patient centrality and improving the quality of care^(12,13)^, added to the complexity and importance of burn cases in children and the absence of instruments for this purpose, it becomes extremely important to validate an instrument for assessing HRQoL in children under 8 years of age who were victims of burns.

## OBJECTIVES

To translate and cross-culturally adapt the BBSIP for Caregivers of Children aged less than 8 years for use in Brazil.

## METHODS

### Ethical aspects

Prior to the study development, the authors of the original instrument were asked for permission and a favorable opinion for cross-cultural adaptation to Brazilian culture. All stages of this study followed ethical aspects, with the study objectives being explained and participants signing the Informed Consent Form before data collection. The research was authorized by the hospital management and approved by the *Universidade Estadual de Londrina* (UEL) Research Ethics Committee.

### Study design, period and location

This is a methodological study characterized by the testing processes of data collection instruments^(14)^. Data collection took place from May 2020 to January 2021, in person and online, through video calls, as this period was marked by the COVID-19 pandemic, which restricted the number of patients treated at the Burn Treatment Center (BTC) outpatient clinic.

The study was carried out at the BTC of a highly complex public university hospital, located in northern Paraná. It is a supplementary unit of UEL, considered a reference hospital in the care of this specialty.

### Population and selection criteria

The study population consisted of four translators, eight evaluators and ten caregivers of children without burn scars in pre-test, and the target population was 34 parents/caregivers of children under 8 years of age with burn scars. Inclusion criteria for the evaluators were proficiency in English and experience in treatment of burn sequels or methodological studies. For the pilot test, parents/caregivers of children under 8 years of age without burn scars and laypeople on the subject were included. For the target population, parents/caregivers of children under 8 years of age with burn scars followed at the BTC outpatient clinic were included.

Exclusion criteria were parents/caregivers who did not accompany their child daily or who had some cognitive or intellectual impairment that impaired communication or memory, which could be considered a factor generating bias, since the instrument refers to actions always carried out in the previous week.

### Study protocol

Cultural adaptation was carried out in seven stages, six stages according to Beaton *et al*.^(11)^, with the inclusion of pilot test with parents/caregivers of children without burn scars to consider the relevance of health literacy in Brazilian Portuguese for adjustments and then applying it to the target population.

**Figure 1:**
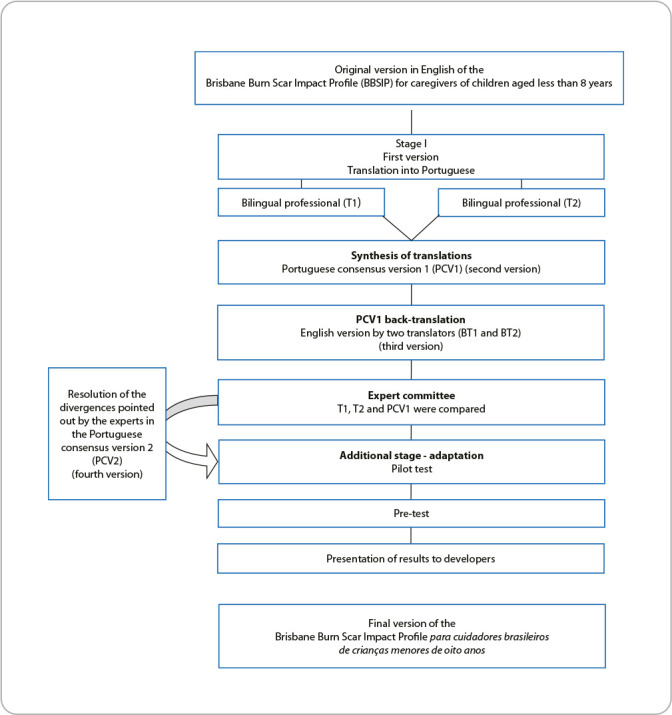
Summary of the cross-cultural adaptation process of the Brisbane Burn Scar Impact Profile for Caregivers of Children aged less than 8 years, Londrina, Paraná, Brazil, 2024

The stages were taken in the following order: initial translation; synthesis of translations; back-translation; expert committee; pilot test; pre-test; and submission of the adapted version for approval by the BBSIP authors (Figure 1).

The first stage involved the translation of the original instrument. The original English version of BBSIP was independently translated into Portuguese by two bilingual translators whose native language was Brazilian Portuguese. One of the translators had technical knowledge on the subject. It should be noted that neither of the translators received any information about the research objectives. Translation version 1 (T1) and translation version 2 (T2) were generated at this stage.

Subsequently, the synthesis of translations was carried out, in which T1 and T2 were analyzed by a third person, who aimed to identify discrepancies, generating a synthesis of translations: Portuguese consensus version 1 (PCV1). Back-translation (BT) into English of PCV1 was carried out by two other independent translators whose native language was English. The instrument authors participated in this stage, giving rise to BT1 and BT2 which, after being analyzed by the researchers, resulted in the final English version (FEV).

The expert committee was made up of eight judges who, in addition to being fluent in English, had experience in burn injury treatment or methodological studies, which they assessed using a Likert scale from 1 to 4, as follows: 1: not relevant; 2: item, to be representative, requires major revision; 3: item, to be representative, requires minor revision; 4: item considered relevant or representative. For each item of the instrument in the four versions (original, T1, T2 and PCV1), semantic, idiomatic, cultural and conceptual equivalences were performed^(15)^. After the assessment carried out by the judges, the researchers resolved the discrepancies found, giving rise to Portuguese consensus version 2 (PCV2).

To assess the instrument clarity, a pilot test was applied to ten parents/caregivers of children under 8 years of age without burn scars, selected at random, in order to verify the literacy of items and general aspects of the Portuguese version. They responded to an adapted five-point Likert-type scale, with “0” being the minimum value (I did not understand anything) and “4” being the maximum value (I understood perfectly and have no doubts). At the end, CVI calculation was applied^(16)^.

For the pre-test, 34 parents/caregivers of children under 8 years old with burn scars participated, based on the recommendations for this stage^(17)^. The last stage involved the preparation of reports of the data generated in the seven-stage process and, subsequently, sending them for consideration by the authors of the original instrument: BBSIP.

### Instruments used

Additionally, sociodemographic and clinical information was collected through printed medical records, the MEDVIEW system and interviews with parents/caregivers.

The version currently being adapted, the BBSIP for caregivers of children under 8 years of age, consists of 58 items, divided into ten groups: overall impact of burn scars (eigh items); itch, pain, discomfort and other sensations (three items); mobility (four items); daily life (nine items); friendships and social interaction (three items); appearance (three items); emotional reactions (seven items); physical symptoms (seven items); parent and family concerns (three items); parental impact (five items). Some items are scored individually and do not belong to any group: item 7 - Sensitivity to light touch or clothing; item 9a - Your child’s daily routine; item 9b - Developing new skills or becoming more independent; item 12 - How bothered has your child been by the appearance of their scars; and item 16 - Did your child have open wounds or sores in their scars^(11)^.

### Analysis of results, and statistics

The collected data were organized in a database in a Microsoft Excel for Windows^®^ spreadsheet and analyzed in SPSS^®^ version 20.0.

The CVI^(16)^ was calculated for the population of judges as follows: the sum of the agreement of items with a score of “3” or “4” divided by the total number of responses, i.e. eight judges. Scores lower than “4” were reviewed.

In the pilot test, for the population of parents/caregivers of children under 8 years old, CVI was calculated by adding the items with a score of “3” or “4” divided by the total number of responses; in this case, ten parents/caregivers. Responses 0, 1 and 2 were considered indicators of insufficient understanding. Values equal to or greater than 0.80 were considered to be an acceptable CVI^(16)^. The internal consistency of the translated and adapted instrument was assessed using Cronbach’s alpha test, considering an acceptable value ≥ 0.70^(18,19)^.

## RESULTS

The BBSIP for Caregivers of Children aged less than 8 years was adapted to Brazilian culture. The Brazilian version was titled “Brisbane Burn Scar Impact Profile (BBSIP-BR) *para cuidadores de crianças abaixo de 8 anos*”.

Adjustments were made by the researchers in one round to reach consensus on T1 and T2, resolving semantic discrepancies and performing PCV1 in the first stage. In the next stage, BT1 and BT2 were analyzed and compared by the researchers, which were quite similar, obtaining the FEV. In this stage, the instrument authors participated by consenting to the version, recommending maintaining the same layout as the original document.

The next stage, expert analysis, was composed of eight judges, being: a nursing professor with a PhD, specialist in instrument validation, at a state university; a nursing professor with a PhD at a state university; a nurse from a university hospital who is a specialist in burns and is a master’s student; a nurse with a PhD in a university hospital who is a specialist in burns; a physiotherapist from a university hospital who is a specialist in burns and is a master’s student; two PhDs in physiotherapy with experience in burns; and a physician from a university hospital with experience in burns.

PCV1 and the other documents in the translation process assessed by the expert committee obtained CVI ≥0.87 for each item, considered satisfactory for meeting semantic, idiomatic, cultural and conceptual equivalences.

All notes were reviewed by two reviewers according to experts’ suggestions (Supplementary File). Grammatical and typing reviews were also performed. After all adjustments, the result was PCV2.

Then, with the application of the pilot test to assess the instrument, of the 58 items that make up the instrument, 35 items presented CVI=1.00, and the remaining 23 items, CVI ≥0.80. It was found that there were no problems regarding the understanding of the instrument by the pilot test population.

In the pre-test stage, the instrument reliability was assessed as adequate, with Cronbach’s alpha = 0.90. Reliability analysis by dimension showed adequate results, except for friendships and social interaction, which obtained an alpha of 0.57 (Table 1), but the exclusion of this item in the calculations resulted in an acceptable value of 0.77.

**Table 1:** Internal consistency of the dimensions of the Brazilian version of the Brisbane Burn Scar Impact Profile for caregivers of children under 8 years old (n=34), Londrina, Paraná, Brazil, 2024

Dimension	Cronbach’s alpha
Overall impact	0.81
Sensory frequency and symptoms	0.82
Mobility	0.85
Daily life	0.73
Friendships and social interaction	0.57
Appearance	0.82
Emotional reactions	0.86
Physical symptoms	0.88
Parental concerns	0.71
Family concerns	0.78

Among the 34 parents/caregivers of children who suffered burns, 5.9% were the father and 94.1% were the mother. Of these, 61.8% were married or in a stable relationship; 32.4% had elementary education; 55.9% had high school education; and 11.7% had higher education. Just over half (52.9%) had paid employment.

Almost all children suffered burn accidents in the home, 58.8% of whom were male and 73.5% were up to 5 years old. Among the causal agents were scalding (52.9%), followed by direct flame (20.5%), contact with hot objects (17.6%), electric shock (5.9%) and chemical shock (2.9%). For 73.5%, the burned body surface was ≤20%.

## DISCUSSION

The cross-cultural adaptation process strictly complied with the recommendations in the literature, even reaching 80% agreement among experts. In order to ensure good understanding by the target population, some grammatical adjustments were necessary to better adapt to the Brazilian context and ensure that it is understood clearly.

The use of experts is essential in this process with psychometric instruments, as in addition to increasing reliability, it clearly highlights the research theme, helping the text to have uniform terms and maintain the linguistic differences of each country^(20)^. Furthermore, the expert committee interdisciplinary composition enriches assessment according to its area of activity^(21)^.

To adapt the method with the inclusion of pilot test^(16)^, it was possible to verify the ease of understanding of each item of the instrument both by the target population and by those who do not experience this condition, considering different levels of education and profiles.

Assessment questionnaires have often been used in various areas of health and, given the scarcity of validated instruments in Brazil that assess HRQoL in caregivers of children with burn scars under 8 years of age, the cross-cultural adaptation of a validated instrument optimizes time and also reduces costs, enabling multicenter studies to compare results^(22)^.

Although there was no need for modifications in our pre-test, it is important to note that this stage is extremely important, as it allows us to arrive at a more consistent preliminary version, and if there are difficulties pointed out by respondents, modifications can be made by the researcher and submitted again to pre-test^(23,24)^.

Regarding psychometrics, the instrument reliability was proven by Cronbach’s alpha. Therefore, items are correlated with each other, except for friendships and social interaction. The researchers decided to maintain this dimension for the final version of the instrument, since it is an essential component for the healthy development of children, including those living with burn scars, which should be considered to assess HRQoL as well as during the collection period. The country was facing the COVID-19 pandemic and in social isolation, restricting access to friendships and interaction, which, therefore, may have influenced the result.

It is worth noting that, for some researchers, any Cronbach’s alpha result greater than 0.60 can be interpreted as satisfactory internal consistency within the framework of the research^(25)^. In this regard, the decision to maintain the dimension for future research is added to confirm the alpha. On the other hand, for some authors, Cronbach’s alpha is considered a positive value if it results between 0.70 and 0.95^(19)^. It is also necessary to reflect on the cultural conditions and the context, not just the translation performed, to support the psychometric properties of a validated instrument, and the method used must guarantee the equivalence of the translated instrument in relation to the original version^(24,26)^.

Concerning accident characteristics, there was a predominance of scalds in children under 5 years old and among males, supporting the results of other studies in the Brazilian scenario^(27-29)^.

### Study limitations

One of the unusual limitations was that the research was conducted during the COVID-19 pandemic and social isolation measures, which restricted the number of patients seen at the BTC outpatient clinic. On the other hand, the possibility of accessing parents/caregivers online did not prevent the study from being conducted.

### Contributions to nursing, health and public policies

The BBSIP for Caregivers of Children aged less than 8 years, translated into Brazilian Portuguese, can be used to assist healthcare professionals in caring for children with burn scars to identify signs and symptoms that may impair the process of caring for skin scars caused by burn injuries and, consequently, assist in implementing measures to improve the quality of life of children, parents and/or family members.

## CONCLUSIONS

The BBSIP-BR for caregivers of children under 8 years of age proved to be reliable for use in Brazil after the cross-cultural adaptation process. The final version was considered appropriate, maintaining semantic, idiomatic and cultural equivalence, and the instrument reliability was proven in pre-test. The translation and adaptation of this instrument provide important contributions to assessing the quality of life of Brazilian children under 8 years of age with burn scars, as it is the first instrument available in Brazil for this age group.

## Data Availability

Not applicable.
