# Supplementary material for: Brisbane Burn Scar Impact Profile for children aged less than 8 years cross-cultural adaptation
Source: Rev Bras Enferm. 2025 Aug 8;78(Suppl 2):e20240153. doi: 10.1590/0034-7167-2024-0153 (PMC12334128; doi:10.1590/0034-7167-2024-0153)
Supplement: Translation

# Translation: Adaptação transcultural do *Brisbane Burn Scar Impact Profile* para menores de 8 anos

**DOI:** 10.1590/0034-7167-2024-0153pt

**Authors:** Francieli Ferreira de Andrade Batista, Rosângela Aparecida Pimenta, Elisângela Flauzino Zampar, Renne Rodrigues, Elza Hiromi Tokushima Anami, Maria Elena Echevarría-Guanilo

**Affiliations:** I Universidade Estadual de Londrina. Londrina, Paraná, Brasil; II Universidade Federal da Fronteira Sul. Chapecó, Santa Catarina, Brasil; III Universidade Federal de Santa Catarina. Florianóplolis, Santa Catarina, Brasil

**Keywords:** Tradução, Estudo de Validação, Criança, Queimaduras, Qualidade de Vida

## Abstract

**Objetivos:**

adaptar transculturalmente o *Brisbane Burn Scar Impact Profile for Caregivers of Children aged less than 8 years* para uso no Brasil.

**Métodos:**

estudo metodológico, seguindo as etapas de tradução, síntese das traduções, retrotradução, comitê de juízes, teste piloto, pré-teste e envio da versão adaptada para aprovação dos autores do instrumento. O Índice de Validade de Conteúdo (IVC) foi utilizado para avaliar a concordância entre os juízes e avaliação da compreensão do instrumento entre os cuidadores. Para o escore global e dimensões do instrumento, foi utilizado o alfa de Cronbach.

**Resultados:**

obteve-se um IVC ≥0,87 para cada item na análise realizada pelo comitê de especialistas, e no teste piloto, IVC ≥0,80. O alfa de Cronbach global foi de 0,94 para todas dimensões, exceto para amizades e interação social (alfa ≥0,70).

**Conclusões:**

a adaptação transcultural apresentou resultados adequados, sustentando a validade da versão traduzida.

## INTRODUÇÃO

O número de casos de queimaduras tem diminuído nos últimos anos em todo o mundo, com uma redução mais significativa no Brasil, enquanto que, em outros países, como a Índia, foi identificado aumento nessa prevalência^(1)^. Ainda assim, as queimaduras na infância continuam sendo um grande desafio para os serviços de saúde e políticas econômicas, pois têm mais da metade dos anos de vida perdidos por incapacidades resultantes dos agravos dos acidentes por queimaduras^(2,3)^. Em geral, as lesões são altamente dolorosas e traumatizantes, que podem produzir um impacto negativo importante tanto no funcionamento físico quanto no psicossocial, levando ao aumento da angústia, ansiedade e trauma, comprometendo a qualidade de vida relacionada à saúde (QVRS) tanto dos pacientes quanto de seus cuidadores^(4-6)^.

A QVRS é um conceito amplamente utilizado na saúde pública, podendo a sua mensuração ser imprecisa na população pediátrica, diante da dificuldade de entendimento e respostas para os itens de avaliação. Por isso, a percepção dos pais/cuidadores é essencial para avaliar a qualidade de vida das crianças^(7)^. A mensuração é essencial para qualificar os ônus provocados pelas cicatrizes de queimaduras em sobreviventes, devendo os profissionais de saúde considerar as perspectivas das vítimas em relação às suas cicatrizes como um importante indicador de qualidade de vida^(8,9)^.

Em razão da complexidade dos cuidados oferecidos às crianças vítimas de queimadura, da diversidade dos casos e subjetividade desse impacto para a QVRS, os instrumentos para avaliação desse construto se mostram imprescindíveis para essa avaliação. No entanto, no Brasil, ainda não há instrumentos específicos validados para esse fim, o que destaca uma lacuna no conhecimento e dificulta a mensuração do impacto das queimaduras na QVRS em crianças menores de 8 anos. Revisão sistemática sobre as propriedades de medição de instrumentos que avaliam a QVRS em crianças aponta o *Brisbane Burn Scar Impact Profile* (BBSIP) como ferramenta adequada para essa população, especialmente quando comparado aos outros dois instrumentos disponíveis na literatura para essa faixa etária^(10)^.

Desse modo, o BBSIP permite avaliar a QVRS em crianças menores de 8 anos, auxiliando na determinação da carga de cicatrização, na funcionalidade, atitudes comportamentais e convivência social após o acometimento desse agravo, bem como permitindo medir com segurança a eficácia das intervenções ao longo do processo de cuidado^(11)^.

Considerando a importância do uso de medidas de resultados relatados pelo paciente para a tomada de decisão clínica, promoção da centralidade do paciente e melhoria da qualidade do atendimento^(12,13)^, somada à complexidade e importância dos casos de queimaduras em crianças e à ausência de instrumentos para este fim, torna-se de extrema importância a validação de instrumento para avaliação da QVRS em crianças menores de 8 anos que foram vítimas de queimaduras.

## OBJETIVOS

Traduzir e adaptar transculturalmente o *BBSIP for Caregivers of Children aged less than 8 years* para uso no Brasil.

## MÉTODOS

### Aspecto éticos

Previamente ao desenvolvimento do estudo, solicitaram-se aos autores do instrumento original a permissão e parecer favorável para a adaptação transcultural para a cultura brasileira. Todas as etapas do estudo seguiram os aspectos éticos, sendo explicados os objetivos do estudo e procedendo à assinatura do Termo de Consentimento Livre e Esclarecido pelos participantes antes da coleta de dados. A pesquisa foi autorizada pela direção do hospital e aprovada pelo Comitê de Ética em Pesquisa da Universidade Estadual de Londrina (UEL).

### Desenho, período e local do estudo

Estudo metodológico caracterizado pelos processos de testagem de instrumentos de coleta de dados^(14)^. A coleta de dados ocorreu no período de maio de 2020 a janeiro de 2021, de maneira presencial e *online*, através de chamadas de vídeo, devido a esse período ter sido marcado pela pandemia de COVID-19, que restringiu o número de pacientes atendidos no ambulatório do Centro de Tratamento de Queimados (CTQ).

O estudo foi realizado no CTQ de hospital universitário público de alta complexidade, localizado na região Norte do estado do Paraná. É um órgão suplementar da UEL, considerado hospital de referência no atendimento dessa especialidade.

### População e critérios de seleção

A população do estudo foi composta por quatro tradutores, oito juízes avaliadores e dez cuidadores de crianças sem cicatrizes de queimaduras no pré-teste, e a população-alvo foram 34 pais/cuidadores de crianças menores de 8 anos com cicatrizes de queimaduras. Os critérios de inclusão para os juízes foram possuir domínio da língua inglesa e experiência na área de tratamento de sequelas de queimaduras ou de estudos metodológicos. Para o teste piloto, pais/cuidadores de criança menores de 8 anos sem cicatrizes de queimaduras e leigos sobre a temática foram incluídos. Para a população-alvo, pais/cuidadores de crianças menores de 8 anos com cicatrizes de queimaduras acompanhadas no ambulatório do CTQ foram incluídos.

Os critérios de exclusão foram pais/cuidadores que não acompanhassem diariamente seu filho ou que apresentassem algum comprometimento cognitivo e intelectual que prejudicasse a comunicação ou a memória, podendo ser considerado um fator gerador de viés, já que o instrumento se refere a ações realizadas sempre na semana anterior.

### Protocolo do estudo

A adaptação cultural foi realizada em sete etapas, seis etapas segundo Beaton *et al*.^(11)^, com a inclusão do teste piloto com pais/cuidadores de crianças sem cicatrizes de queimaduras para considerar a pertinência quanto ao letramento em saúde na língua portuguesa do Brasil para ajustes e após aplicar para a população-alvo.

As etapas se deram na seguinte ordem: tradução inicial; síntese das traduções; retrotradução; comitê de especialistas; teste piloto; pré-teste; e envio da versão adaptada para aprovação dos autores do BBSIP (Figura 1).

**Figura 1:** Síntese do processo de adaptação transcultural do *Brisbane Burn Scar Impact Profile for Caregivers of Children aged less than 8 years,* Londrina, Paraná, Brasil, 2024

A primeira etapa correspondeu à tradução do instrumento original. A versão original em inglês do BBSIP foi traduzida para o português de maneira independente por dois tradutores bilíngues que possuíam como língua nativa o português do Brasil. Um dos tradutores detinha conhecimento técnico acerca do tema. Ressalta-se que nenhum dos tradutores recebeu qualquer informação acerca dos objetivos da pesquisa. A tradução versão 1 (T1) e tradução versão 2 (T2) foram geradas nessa etapa.

A seguir, realizou-se a síntese das traduções, em que as versões T1 e T2 foram analisadas por uma terceira pessoa, que teve o intuito de identificar discrepâncias, sendo gerada uma síntese das traduções: versão português consenso 1 (VPC1). A retrotradução (R) para a língua inglesa da versão VPC1 foi realizada por outros dois tradutores independentes que tinham como língua materna o inglês. Participaram dessa etapa os autores do instrumento, dando origem às R1 e R2 que, após serem analisadas pelas pesquisadoras, deram resultado à versão inglês final (VIF).

O comitê de especialistas foi constituído por oito juízes que, além de possuírem domínio da língua inglesa, possuíam experiência na área de tratamento de sequelas de queimaduras ou de estudos metodológicos que avaliaram por meio de uma escala Likert de 1 a 4, sendo: 1: não relevante; 2: item, para ser representativo, necessita de grande revisão; 3: item, para ser representativo, precisa de pequena revisão; 4: item considerado relevante ou representativo. Para cada item do instrumento nas quatro versões (original, T1, T2 e VPC1), foram realizadas as equivalências semântica, idiomática, cultural e conceitual^(15)^. Após a avaliação realizada pelos juízes, as pesquisadoras resolveram as divergências encontradas, dando origem à versão português consenso 2 (VPC2).

Para avaliar a clareza do instrumento, aplicou-se o teste piloto com dez pais/cuidadores de crianças menores de 8 anos sem cicatrizes de queimadura, selecionados de forma aleatória, a fim de verificar o letramento dos itens e aspectos gerais da versão português do instrumento. Responderam uma escala do tipo Likert adaptada de cinco pontos, sendo “0” o valor mínimo (não entendi nada) e “4” o valor máximo (entendi perfeitamente e não tenho dúvidas). Ao final, foi aplicado o cálculo do IVC^(16)^.

Para o pré-teste, participaram 34 pais/cuidadores de crianças menores de 8 anos com cicatrizes de queimaduras, com base nas recomendações para esta etapa^(17)^. A última etapa se deu com a confecção de relatórios dos dados gerados no processo das sete etapas e, posteriormente, o envio para apreciação dos autores do instrumento original: BBSIP.

### Instrumentos utilizados

Adicionalmente, foram coletadas informações sociodemográficas e clínicas por meio de prontuários impressos, sistema MEDVIEW e entrevista com os pais/cuidadores.

A versão em processo de adaptação, o BBSIP para cuidadores de crianças menores de 8 anos, é composta por 58 itens, divididos em dez grupos: impacto geral (oito itens); frequência sensorial (três itens); mobilidade (quatro itens); vida diária (nove itens); amizades e interação social (três itens); aparência (três itens); reações emocionais (sete itens); sintomas físicos (sete itens); preocupação dos pais (três itens); impacto dos pais (cinco itens). Alguns itens são pontuados de forma individual, não pertencendo a nenhum grupo: item 7 - Sensibilidade ao toque leve ou roupas; item 9a - A rotina diária do seu filho; item 9b - Desenvolver novas habilidades ou tornar-se mais independente; item 12 - O quanto seu filho ficou incomodado com o aparecimento de suas cicatrizes; e o item 16 - Você tinha feridas abertas em suas cicatrizes^(11)^.

### Análise dos resultados e estatística

Os dados coletados foram organizados em banco de dados em planilha do *software Microsoft Excel for Windows* ^®^ e analisados no programa SPSS^®^, versão 20.0.

Calculou-se o IVC^(16)^ para a população de juízes da seguinte forma: a soma de concordância dos itens com pontuação “3” ou “4” dividida pelo número total de respostas, ou seja, oito juízes. As pontuações menores de “4” foram revisadas.

No teste piloto, para a população de pais/cuidadores de crianças menores de 8 anos, calculou-se o IVC por meio da soma dos itens com pontuação “3” ou “4” dividida pelo número total de respostas; no caso, dez pais/cuidadores. As respostas 0, 1 e 2 foram consideradas como indicadores de uma compreensão insuficiente. Consideraram-se valores iguais ou superiores a 0,80 para um IVC aceitável^(16)^. A consistência interna do instrumento traduzido e adaptado foi avaliada, aplicando-se o teste de alfa de Cronbach considerando-se como valor aceitável ≥ 0,70^(18,19)^.

## RESULTADOS

O *BBSIP for Caregivers of Children aged less than 8 years* foi adaptado para a cultura brasileira. A versão brasileira recebeu o título de “*Brisbane Burn Scar Impact Profile* (BBSIP-BR) para cuidadores de crianças abaixo de 8 anos”.

Ajustes foram realizados pelos pesquisadores, em uma rodada, para o consenso das T1 e T2, resolvendo discrepâncias semânticas e realizando a VPC1 na primeira etapa. Na etapa seguinte, de retrotradução, R1 e R2 foram analisadas e comparadas pelas pesquisadoras, que estavam bastante semelhantes, obtendo-se a VIF. Nesta etapa, os autores do instrumento participaram consentindo com a versão, recomendando manter o mesmo *layout* do documento original.

A etapa seguinte, de análise dos especialistas, foi composta por oito juízes, sendo: um professor doutor do curso de enfermagem, especialista em validação de instrumentos, de universidade estadual; uma professora doutora do curso de enfermagem de universidade estadual; uma enfermeira de hospital universitário especialista em queimados e mestranda; uma enfermeira doutora de hospital universitário especialista em queimados; uma fisioterapeuta de hospital universitário especialista em queimados e mestranda; duas doutoras em fisioterapia com experiência em queimados; e um médico de hospital universitário com experiência em queimados.

A VPC1 e os demais documentos do processo de tradução avaliada pelo comitê de especialistas obtiveram IVC ≥0,87 para cada item, considerado satisfatório para atender às equivalências semântica, idiomática, cultural e conceitual.

Revisão de todas as anotações foi realizada por dois revisores de acordo com as sugestões dos especialistas (Arquivo Suplementar). Também houve revisão gramatical e de digitação. Após todos os ajustes, resultou na VPC2.

Em seguida, com a aplicação do teste piloto para avaliação do instrumento, dos 58 itens que compõem o instrumento, 35 itens apresentaram IVC=1,00, e os 23 itens restantes, IVC ≥0,80. Constatou-se que não houve problemas quanto à compreensão do instrumento pela população do teste piloto.

Na etapa de pré-teste, avaliou-se a confiabilidade do instrumento como adequada, com alfa de Cronbach = 0,90. A análise da confiabilidade por dimensão apresentou resultados adequados, exceto amizades e interação social, que obteve um alfa de 0,57 (Tabela 1), mas a exclusão deste item nos cálculos resultou no valor aceitável de 0,77.

**Tabela 1:** Consistência Interna das dimensões da versão brasileira do *Brisbane Burn Scar Impact Profile* para cuidadores de crianças abaixo de 8 anos (n=34), Londrina, Paraná, Brasil, 2024

Dimensão	Alfa de Cronbach
Impacto geral	0,81
Frequência sensorial e sintomas	0,82
Mobilidade	0,85
Vida diária	0,73
Amizades e interação social	0,57
Aparência	0,82
Reações emocionais	0,86
Sintomas físicos	0,88
Preocupação dos pais	0,71
Preocupações familiares	0,78

Entre os 34 pais/cuidadores das crianças que sofreram queimaduras, 5,9% eram o pai, e 94,1%, a mãe. Desses, 61,8% eram casados ou em união estável; 32,4% tinham ensino fundamental; 55,9% tinham ensino médio; e 11,7% tinham ensino superior. Pouco mais da metade (52,9%) possuía trabalho remunerado.

Quase a totalidade das crianças sofreu os acidentes por queimaduras no ambiente doméstico, sendo 58,8% do sexo masculino e 73,5% com até 5 anos de idade. Entre os agentes causais, houve escaldadura (52,9%), seguida por chama direta (20,5%), contato com objetos quentes (17,6%), choque elétrico (5,9%) e químico (2,9%). Para 73,5%, a superfície corpórea queimada foi ≤20%.

## DISCUSSÃO

O processo de adaptação transcultural cumpriu rigorosamente o preconizado na literatura, mesmo alcançando 80% de concordância entre os especialistas. Com o objetivo de garantir uma boa compreensão pela população-alvo, alguns ajustes gramaticais se fizeram necessários para melhor adaptação ao contexto brasileiro e garantir que esta seja entendida com clareza.

A utilização de especialistas é fundamental nesse processo com instrumentos psicométricos, pois além de ampliar a confiabilidade, evidencia de forma clara a temática da pesquisa, colaborando para que o texto possua termos uniformes e mantenha as diferenças linguísticas de cada país^(20)^. Além disso, a composição interdisciplinar do comitê de especialistas enriquece a avaliação de acordo com sua área de atuação^(21)^.

Para adaptação do método com a inclusão do teste piloto^(16)^, foi possível verificar a facilidade de compreensão de cada item do instrumento tanto pela população-alvo quanto pela que não vivencia este agravo, contemplando diferentes níveis de escolaridade e perfis.

Frequentemente, questionários de avaliação têm sido utilizados em diversas áreas da saúde e, diante da escassez de instrumentos validados no Brasil que avaliam QVRS em cuidadores de crianças com cicatrizes de queimaduras menores de 8 anos, a adaptação transcultural de um instrumento validado otimiza o tempo e ainda diminui os gastos, possibilitando estudos multicêntricos para confrontação de resultados^(22)^.

Ainda que em nosso pré-teste não houvesse a necessidade de modificações, ressalta-se que tal etapa é de suma importância, pois permite chegar a uma versão preliminar mais condizente, e se houver dificuldades apontadas pelos respondentes, as modificações podem ser realizadas pelo pesquisador e submetidas novamente ao pré-teste^(23,24)^.

No que tange à psicometria, foi comprovada a confiabilidade do instrumento pelo alfa de Cronbach. Sendo assim, os itens estão correlacionados entre si, exceto para amizades e interação social. Os pesquisadores decidiram pela manutenção dessa dimensão para a versão final do instrumento, visto ser um componente essencial para o desenvolvimento saudável da criança, inclusive para aquelas que convivem com cicatrizes de queimaduras, que deve ser considerado para avaliar a QVRS, bem como durante o período da coleta. O país estava enfrentando a pandemia de COVID-19 e em isolamento social, restringindo o acesso às relações de amizade e interação que, por conseguinte, pode ter influenciado o resultado.

Vale destacar que, para alguns pesquisadores, qualquer resultado do alfa de Cronbach superior a 0,60 pode ser interpretado como uma consistência interna satisfatória no enquadramento da pesquisa^(25)^. Neste sentido, soma-se a decisão na manutenção da dimensão para futuras pesquisas para a confirmação do alfa. Por outro lado, para alguns autores, o alfa de Cronbach é considerado um valor positivo se resultar entre 0,70 e 0,95^(19)^. É necessário também refletir sobre as condições culturais e o contexto, não apenas a tradução realizada, para sustentar as propriedades psicométricas de um instrumento validado, e o método utilizado deve garantir a equivalência do instrumento traduzido em relação à versão original^(24,26)^.

Quanto às características dos acidentes, houve predominância de escaldaduras em menores de 5 anos e entre o sexo masculino, corroborando o resultado de outros estudos no cenário brasileiro^(27-29)^.

### Limitações do estudo

Uma das limitações inusitadas foi a realização da pesquisa durante a pandemia de COVID-19 e as medidas de isolamento social, que restringiram o número de pacientes atendidos no ambulatório do CTQ. Por outro lado, a possibilidade de acesso aos pais/cuidadores na modalidade *online* não impediu a realização do estudo.

### Contribuições para às áreas da enfermagem, saúde e políticas públicas

O *BBSIP for Caregivers of Children aged less than 8 years*, traduzido para o português brasileiro, pode ser utilizado para auxiliar profissionais de saúde no atendimento de crianças com cicatrizes de queimaduras para identificação de sinais e sintomas que podem prejudicar o processo de cuidado da cicatriz de pele ocasionado por lesões de queimaduras e, por conseguinte, auxiliar na implementação de medidas para melhorar a qualidade de vida da criança, pais e/ou familiares.

## CONCLUSÕES

O BBSIP-BR para cuidadores de crianças abaixo de 8 anos se mostrou confiável para ser utilizado no Brasil após o processo de adaptação transcultural. A versão final foi considerada apropriada, mantendo a equivalência semântica, idiomática e cultural, sendo comprovada a confiabilidade do instrumento na realização do pré-teste. A tradução e adaptação desse instrumento preveem importantes contribuições para avaliar a qualidade de vida de crianças brasileiras menores de 8 anos de idade com cicatrizes de queimaduras, pois é o primeiro instrumento disponível no Brasil para esse grupo etário.

## Data Availability

Não se aplica.
